# Supplementary material for: Detection of infiltrating fibroblasts by single-cell transcriptomics in human kidney allografts
Source: PLoS One. 2022 Jun 3;17(6):e0267704. doi: 10.1371/journal.pone.0267704 (PMC9165878; doi:10.1371/journal.pone.0267704)
Supplement: S1 File — (ZIP) [file pone.0267704.s001.zip › PONE-D-21-17912_R3__Supporting_Information_____/S2_table.pdf]

S2 Table. Cell-type-specific expression of the X chromosome marker gene XIST

Cell type	HK	Cell type	AK1	Cell type	AK2
PT	0	PT	0.026925	PT	0.063701
PG	3.868948	PG	0.018473	PG	3.491369
LH	9.334275	CD	0.029711	CD	4.220906
CD	5.450415	IC.A	0	IC.A	3.703303
IC.A	4.310801	FB1	3.530406	FB4	4.782507
FB2	2.440544	FB4	0.675691	AVR	3.926241
FB3	3.04712	AVR	0.290284	vSMC	3.18347
AVR	5.393421	vSMC	0	PC	3.794725
DVR	4.014057	PC	0	TC.CTC	0
vSMC	3.586586	TC.CTC	3.808262	NK	0
PC	4.797342	MAC	4.314509	MONO	0.017436
TC.CTC	2.325882	DC	3.854761	MAC	0
MAC	5.780216	BC	9.149131	DC	0.029137
DC	3.219575			BC	0
				PLASMA	0
				MAST	0

- Cell types with greater than 10 cells are shown
- Numbers represent TPM values
- AK1: Donor - male; Recipient - female
- AK2: Donor - female; Recipient - male

Immune cells

Absolute expression values in transcript per million (TPM) of XIST gene. XIST gene produces X-inactive specific transcript (Xist) RNA, a non-coding RNA that is a major effector of the X chromosome inactivation. The Xist RNA is expressed only on the inactive chromosome and not on the active chromosome. Males (XY), who have only one X chromosome that is active, do not express it. Females (XX), who have one active and one inactive X chromosome, express it. In healthy kidney HK (female kidney), all the cells in the kidney express XIST and none express the Y chromosome markers. In AK1 biopsy (male donor and female recipient), all the kidney parenchymal cells express Y chromosome markers whereas all the recipient-derived immune infiltrating cells express XIST. In AK2 biopsy (female donor and male recipient), all the kidney parenchymal cells express XIST whereas all the recipient-derived immune infiltrating cells express the Y chromosome markers. Thus, in the three biopsies, the X and Y chromosome markers are expressed as expected. Interestingly, the fibroblasts in AK1 biopsy (male donor and female recipient), expressed XIST, proving that these were recipient derived.
