# Supplementary material for: Detection of infiltrating fibroblasts by single-cell transcriptomics in human kidney allografts
Source: PLoS One. 2022 Jun 3;17(6):e0267704. doi: 10.1371/journal.pone.0267704 (PMC9165878; doi:10.1371/journal.pone.0267704)
Supplement: S1 File — (ZIP) [file pone.0267704.s001.zip › PONE-D-21-17912_R3__Supporting_Information_____/S5_fig.pdf]

S5 Fig. Comparison of kidney fibroblasts with skin fibroblasts

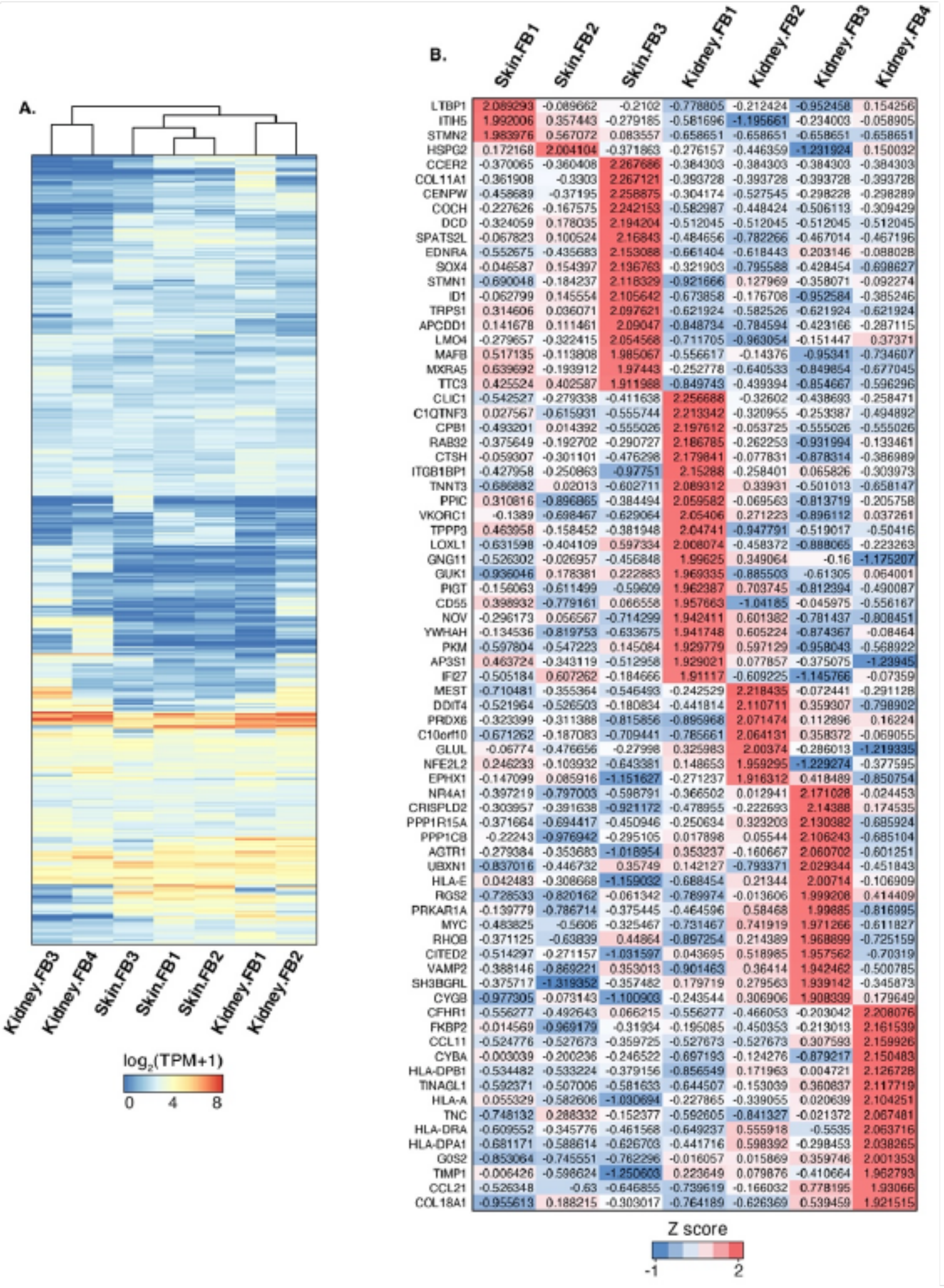

We compared the gene expression profiles of kidney fibroblasts with that of skin fibroblasts. We have reported 3 fibroblast subtypes by scRNA-seq of human skin from 5 patients with atopic dermatitis and 7 healthy control subjects *(He H et al. Single-cell transcriptome analysis of human skin identifies novel fibroblast subpopulation and enrichment of immune subsets in atopic dermatitis. J Allergy Clin Immunol. 2020 Jun;145(6):1615-1628).* In order to compare the expression profile of FBs in the allograft kidney with the FBs in skin, we selected union of top 100 genes expressed in each FB population and performed unsupervised clustering as seen in the heatmap (Panel A). Next, we analyzed the most abundant and differentially expressed genes in all the FBs using the 'Z score' (Panel B).
