# Supplementary material for: Detection of infiltrating fibroblasts by single-cell transcriptomics in human kidney allografts
Source: PLoS One. 2022 Jun 3;17(6):e0267704. doi: 10.1371/journal.pone.0267704 (PMC9165878; doi:10.1371/journal.pone.0267704)
Supplement: S1 File — (ZIP) [file pone.0267704.s001.zip › PONE-D-21-17912_R3__Supporting_Information_____/S3_fig.pdf]

S3 Fig. Prediction scores for cell clusters when mapped to a kidney reference dataset

A

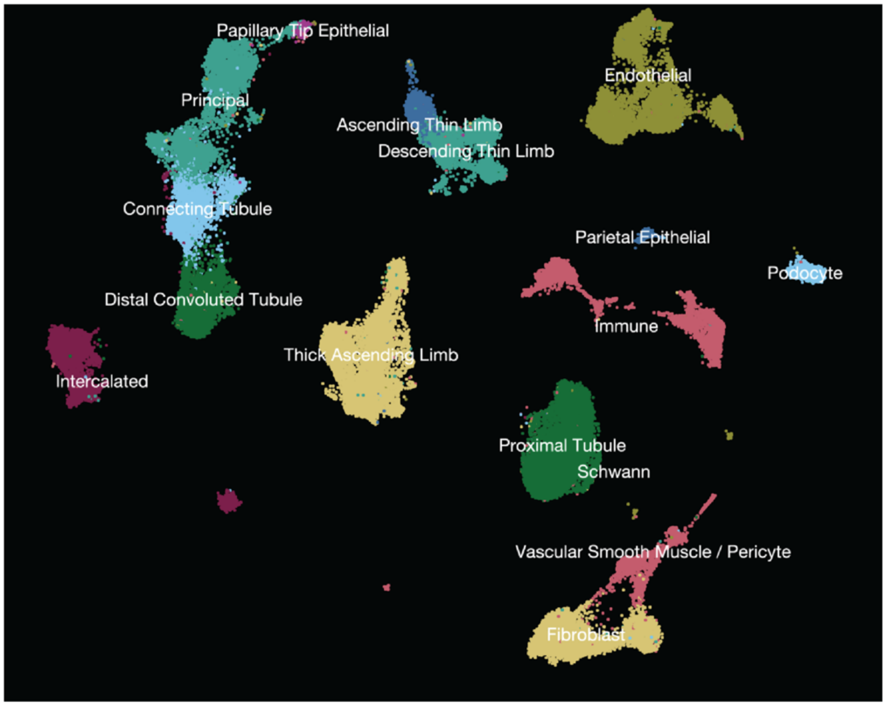

B

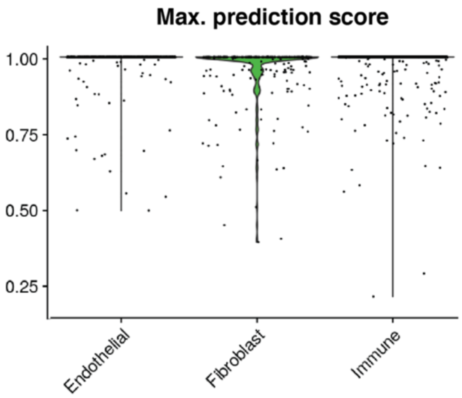

(A). Reference dataset consisting of 64,693 kidney cells generated in the Human Biomolecular Atlas Program (HuBMAP) and the Kidney Precision Medicine Project (KPMP) *(Lake BB et al. An atlas of healthy and injured cell states and niches in the human kidney, bioRxiv. 2021:2021.07.28.454201. doi: 10.1101/2021.07.28.454201)*. The reference dataset represents 21 samples across 13 patient donors.

(B). We used 'Azimuth' *(Hao Y et al. Integrated analysis of multimodal single-cell data. Cell. 2021;184(13):3573-87)* to map our data to an annotated reference dataset. Most of the cells from our dataset mapped with high prediction score to the reference data; noticeably, as shown in the violin plot, fibroblasts mapped with very high prediction score, confirming the authenticity of our original annotation of fibroblasts.
